# Laser coagulation and hemostasis of large diameter blood vessels: effect of shear stress and flow velocity

**DOI:** 10.1038/s41598-022-12128-1

**Published:** 2022-05-19

**Authors:** Nitesh Katta, Daniel Santos, Austin B. McElroy, Arnold D. Estrada, Glori Das, Mohammad Mohsin, Moses Donovan, Thomas E. Milner

**Affiliations:** 1 University of California at Irvine, Irvine, CA USA; 2 The University of Texas at Austin, Austin, TX USA; 3 University of Connecticut, Storrs, CT USA; 4 Pennsylvania State University, State College, PA USA

**Keywords:** Biomedical engineering, Biological physics

## Abstract

Photocoagulation of blood vessels offers unambiguous advantages to current radiofrequency approaches considering the high specificity of blood absorption at available laser wavelengths (e.g., 532 nm and 1.064 µm). Successful treatment of pediatric vascular lesions, such as port-wine stains requiring microvascular hemostasis, has been documented. Although laser treatments have been successful in smaller diameter blood vessels, photocoagulation of larger sized vessels is less effective. The hypothesis for this study is that a primary limitation in laser coagulation of large diameter blood vessels (500–1000 µm) originates from shear stress gradients associated with higher flow velocities along with temperature-dependent viscosity changes. Laser (1.07 µm) coagulation of blood vessels was tested in the chicken chorio-allantoic membrane (CAM). A finite element model is developed that includes hypothetical limitations in laser coagulation during irradiation. A protocol to specify laser dosimetry is derived from OCT imaging and angiography observations as well as finite element model results. Laser dosimetry is applied in the CAM model to test the experimental hypothesis that blood shear stress and flow velocity are important parameters for laser coagulation and hemostasis of large diameter blood vessels (500–1000 µm). Our experimental results suggest that shear stress and flow velocity are fundamental in the coagulation of large diameter blood vessels (500–1000 µm). Laser dosimetry is proposed and demonstrated for successful coagulation and hemostasis of large diameter CAM blood vessels.

## Introduction

Laser-based microvascular hemostasis was first demonstrated in 1981 under the principle of selective photothermolysis^1^. Subsequently, additional work^2–7^ was carried out to provide laser dosimetry to optimize various parameters (e.g., wavelength, pulse duration, fluence) to achieve hemostasis. Port wine stains (PWSs) are vascular malformations^7–10^ and were one of the first target applications of selective photothermolysis, where a vascular-specific wavelength irradiates blood and is differentially absorbed by hemoglobin in blood.

Conventional surgical procedures still rely on radio frequency (RF) electrocautery for coagulation and hemostasis^11,12^, where collateral nonspecific damage zones are as large as a few millimeters (much larger than the size of targeted blood vessels). These RF tools operate at higher input energy/power and are utilized for soft-tissue cutting and represent commonly employed tools for most surgical procedures. Laser coagulation of blood vessels, however, has limited use in the operating room (OR) for coagulation/hemostasis during conventional surgery, outside of dermal/oral/dental soft tissue surgical applications^13–16^. One possible reason for underutilization of lasers in the OR relates to the selection of laser dosimetry that offers good hemostasis while providing relatively comparable tissue removal rates to conventional cautery tools. For example, because the emission wavelength (2.94 µm) of Er:YAG laser radiation coincides with an absolute peak in water absorption^17^, tissue ablation is highly energy efficient^18^; however, Er:YAG lasers are not used in general surgery (outside of skin cosmetic resurfacing) and are not a viable candidate for vascular coagulation. Alternatively, diode lasers have been shown to perform well for coagulation of blood vessels through hot tip hemostasis^15^ approach but suffer from nonspecific thermal damage. Pulsing strategies have been demonstrated ex vivo to reduce thermal damage and modeling results have shown laser sealing of blood vessels to have adequate mechanical strength^19,20^. Recently, dual-wavelength approaches^21,22^ have been successfully tested by demonstrating high tissue removal rates through pulsed laser irradiation, while coagulation is achieved through continuous (CW) irradiation^21,23^.

Second, another challenge for laser-based vascular hemostasis during surgery is the presence of large diameter (500–1000 µm) blood vessels. In this manuscript we refer to blood vessels between 500 µm and 1 mm as “large blood vessels”. Histopathological and other imaging studies have shown that PWS dilated blood vessel sizes vary between patients in the range of 200-750 μm^24–26^. PWS lesions often consist of slower blood flow rates, with most blood vessels being dilated venules, unlike blood vessels encountered during surgery in the OR. Limitations of selective photothermolysis are also recognized in treating PWS lesions in adults compared to infants who possess smaller diameter blood vessel capillary networks (PWS is a congenital disorder)^8,10^. Similarly, during surgery, typical target blood vessel networks are more pronounced in terms of size and flow rates. For these cases, vascular specific wavelengths for laser hemostasis during surgery potentially offer many improvements over existing electrocautery tools. Specifically, infrared laser sources at a wavelength of approximately 1 µm allow for fiber delivery; deeper penetration in blood potentially allows for hemostasis in millimeter-diameter blood vessels (in comparison to 100 s of microns in PWS) while still offering high specificity to blood optical absorption; and deep (centimeters) tissue penetration^27,28^. Hence, there is a need for the development of laser irradiation protocols and dosimetry for vascular-specific hemostasis in vascular networks with larger blood vessels. In this study, a state-of-the-art ytterbium (Yb) fiber laser (1.07 µm) was used to produce hemostasis in millimeter-sized blood vessels in the chicken chorio-allantoic membrane (CAM) model^29–31^. CAMs allow for quick iterative studies due to a relatively simple preparation protocol and short gestation time and are attractive for hemostasis studies in relatively large diameter blood vessels (millimeters) with high flow rates^32^. In addition, for the study reported here, due to the absence of an epithelial layer with vascular networks being formed on the membrane, confounding complexities, including the presence of additional chromophores (e.g., melanin), were not present, simplifying the experiments (CAM protocols, benchtop setup, vascular analysis and photothermolysis estimations for laser dosimetry at 1.07 µm can be found in supplementary Sects. 1–3). A finite element model (FEM) was developed in COMSOL software to investigate the limitations of the current theory of photothermolysis of large blood vessels. Experimental results are presented that illustrate the importance of visco-velocity changes that occur during laser irradiation of large diameter blood vessels. Initially, laser dosimetry derived from the conventional theory of selective photothermolysis was applied to CAM vessels, resulting in failed coagulation of larger vessels. By increasing radiant energy input based on FEM, laser dosimetry is proposed and demonstrated for successful coagulation of large blood vessels.

## Results and discussion

**Figure 1:**
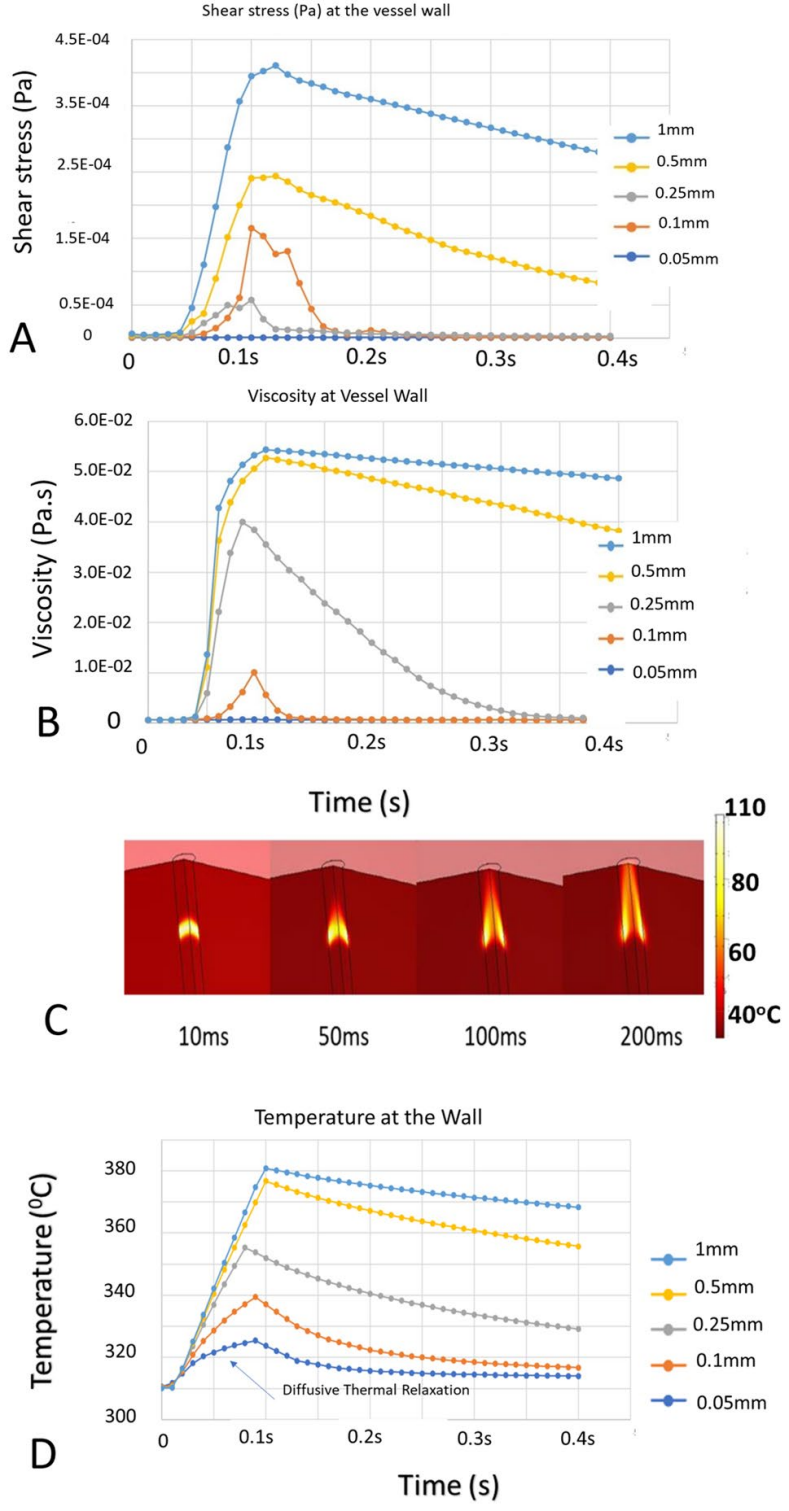
(**A**) Shear stress (Pa) at the vessel luminal wall for five blood vessel lumen diameters (0.05–1 mm) in response to pulsed laser (1.07 µm) irradiation (80 ms, 300 W average power). Shear stress increase is significantly higher post irradiation in the larger blood vessel case suggesting higher chances of coagulum breaking free from the wall as observed experimentally (Fig. 3). Irradiation is simulated as a flat top laser fluence from 0.05 to 0.1 s. (**B**) Viscosity (Pa.s) at the vessel luminal wall for five blood vessel sizes (0.05–1 mm) in response to pulsed laser (1.07 µm) irradiation (80 ms, 300 W average power). (**C**) Temperature profile within a blood vessel (1 mm diameter lumen) modified by blood flow dynamics (blood flowing from bottom to top). Heat at the walls persists while thermal energy is carried away at the center of the blood vessel. For large blood vessel diameters, heat transfer is dominated by blood flow over conduction. (**D**) Temperature relaxation observed during irradiation of smaller diameter blood vessels reduces viscosity whereas the viscosity in larger vessels remains elevated for longer times. The effect is consistent with an experimental result (Fig. 3, Panel C), just before the coagulum attached to the vessel wall is dislodged.

Shear stress FEM computations at the vessel wall indicate that peak stress increases during and immediately after laser irradiation (Fig. 1A). Computed changes in visco-elastic properties show significant localization of increased shear stress near the walls of larger blood vessels. The observed shear stress increase near the vessel wall can be attributed to two effects: (1) an increase in viscosity with increasing temperature (Fig. 1B) (modeled as an interpolated look-up table with data from prior literature^33^) and density changes associated with the coagulum that grows from the central lumen toward the vessel wall, resulting in a higher shear force. Although heat transfer through convective flow is observed (Fig. 1D) in larger vessels as opposed to smaller vessels, Arrhenius computations yielded values of Ω = 1 (corresponding to coagulation) in all cases. The results suggest that relying solely on an Arrhenius damage integral computation to predict a successful hemostatic response while ignoring the mechanical shearing effects of the coagulum, especially at the vessel wall, can introduce erroneous conclusions and artifacts. The importance of mechanical shearing effects at the vessel wall is especially important at higher blood flow velocities in larger diameter vessels.

Considering the dynamic flow-convective properties in larger blood vessels and limitations in explosive vapor bubble formation, an alternative laser-irradiation protocol was developed to increase the initial energy input into larger diameter blood vessels. First, a long duration pulse was introduced to modify the viscosity-dependent flow dynamics in the blood vessel. Long pulse durations have been previously recommended^34–37^ for effective thermolysis when using intense infrared sources, such as those utilized in this study. Considering some of the effects observed in response to long pulse irradiation, including shear stress, velocity and viscosity changes, we hypothesized that subsequent shorter duration laser pulses of reduced energy may allow for successful photocoagulation of large blood vessels. An analog to possibly better understand the laser irradiation protocol proposed here is mechanically reducing the flow/shear stress dynamics by first pinching the vessel (i.e., long pulse laser irradiation) before applying a radiant energy pulse to coagulate the vessel^38,39^. Instead of mechanically constraining the vessel to change the shear/velocity dynamics for successful hemostasis, a long duration pulse changes these parameters as if the vessel was pinched (Fig. 1) before subsequent shorter pulses perform successful hemostasis. The mechanical strain applied to larger blood vessels while pinching using electrocautery tools often results in tissue welding to the electrodes, resulting in rupture when the tool is pulled away. COMSOL simulations highlighted the limitations of employing long laser pulses that were possible with available fluence rates at pulse durations of approximately 100–200 ms (Fig. 1). In blood vessels with smaller diameters, thermal relaxation was observed (Fig. 1D). In larger blood vessels, heat is primarily carried away from the irradiation site through fast convective blood flow (Fig. 1C,D). FEM suggests that a vascular system exposed to an initial high-energy light pulse is modified and can then better satisfy the conditions of the theory of selective photothermolysis due to the modified flow dynamics.

### Limitations of laser coagulation in large vessels

**Figure 2:**
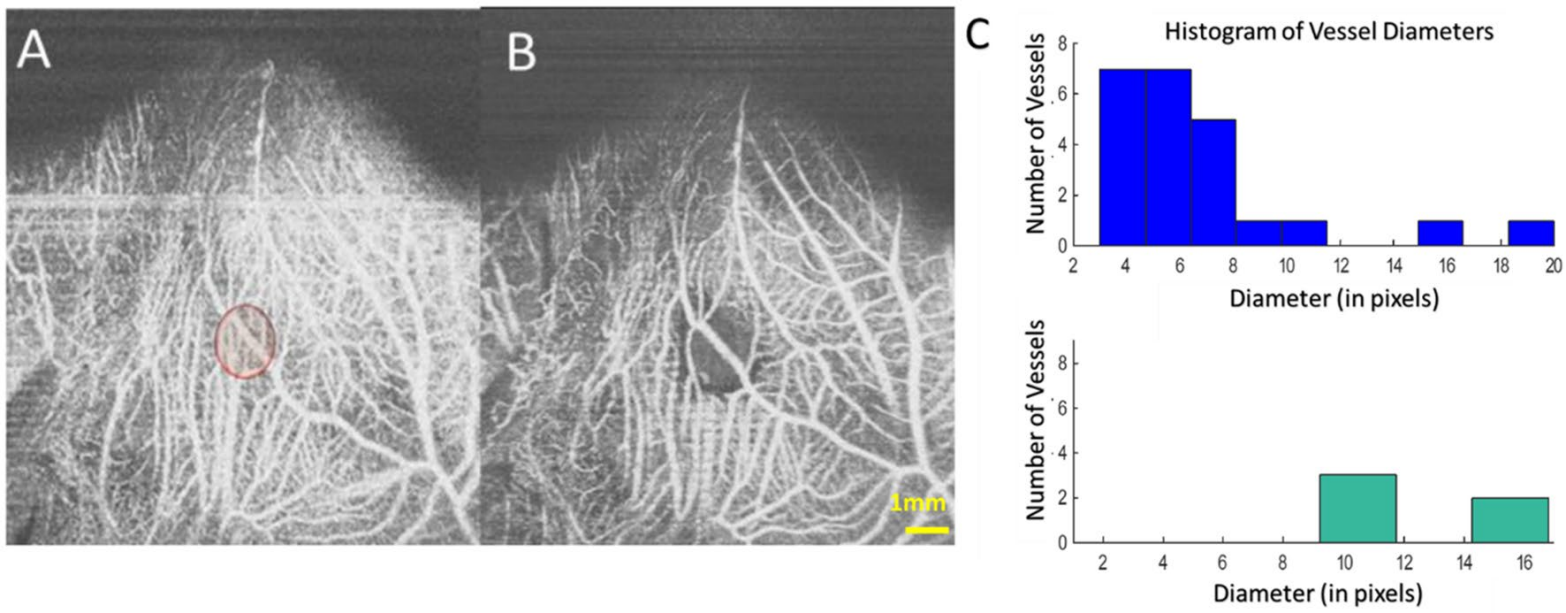
Coagulation of a CAM vasculature showing flow intact in the larger diameter vessels compared to the smaller microvasculature. Histogram analysis of blood vessel size distribution showing hemostasis only occurred in smaller vessels (right). Laser dosimetry: 7.7 J, 25 ms. Spot size 3 mm. Scale Bar is 1 mm. In the 512 × 512 enface image, each pixel corresponds to approximately 20 µm.

After application of the derived laser irradiation protocol (Fig. 2, green bar plot), OCT angiography images recorded pre- and post- laser irradiation (Fig. 2) showed that larger blood vessels (greater than 200 µm) were not coagulated (additional examples are provided in supplementary Figs. S5, S7, S8, S9).

**Figure 3:**
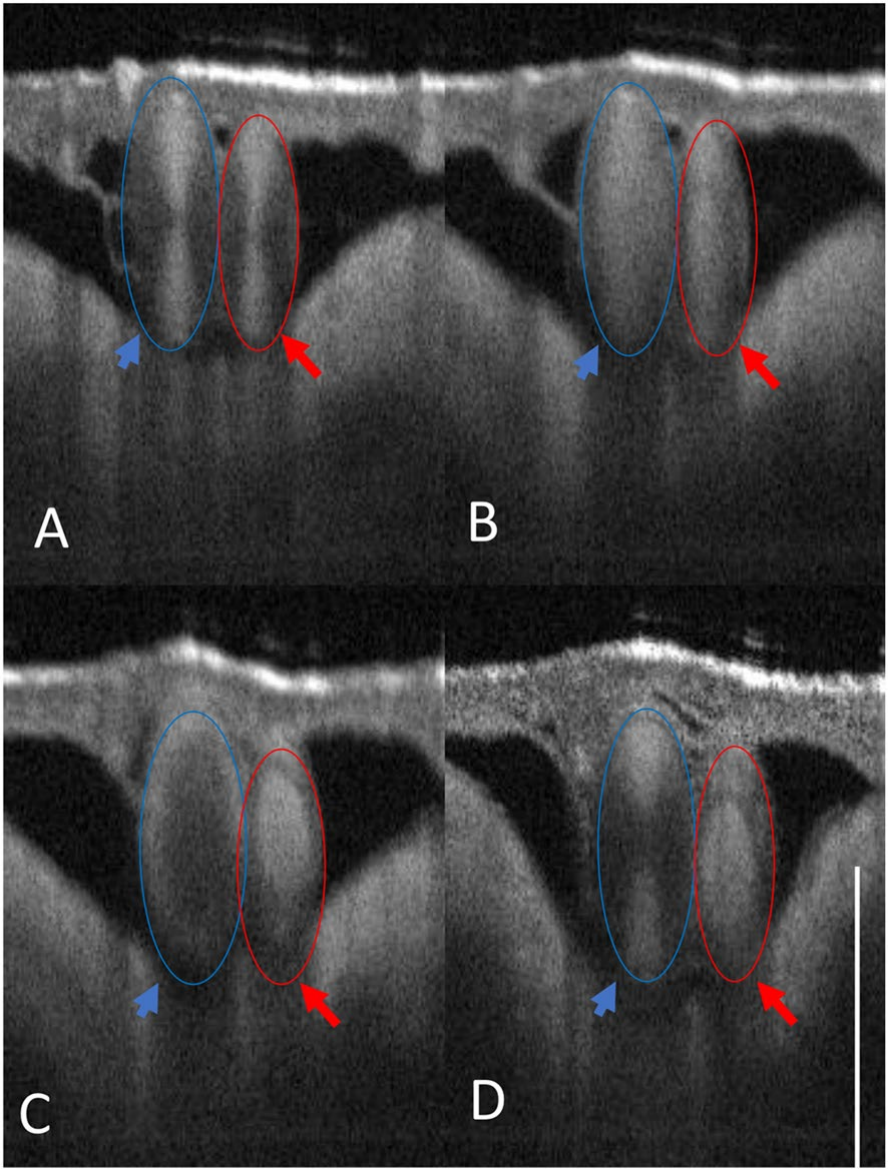
OCT B-scan image of CAM showing an arteriole (red)/venule (blue) pair irradiated at the same fluence rate. (**A**) Initial OCT B-scan before laser irradiation. (**B**) Coagulum formation observed in both arterioles and venules. (**C**) In the arteriole, the coagulum holds, resulting in hemostasis, while in the venule, the coagulum breaks free near the wall. (**D**) Blood flow is restored in the venule where the coagulum breaks from the vessel wall (Panel **C**). Please see supplementary Fig. S10 for enface images collected from this experiment. Scale bar 500 µm. These arteriole/venule pairs were chosen on the CAM model appropriately sized at Day 7 with direct connection to major CAM vessels.

We observed that arterioles more readily coagulated (red dotted line in Panel A, Fig. 3), while flow in venules (blue arrow line, Panel D, Fig. 3) frequently persisted after laser irradiation, similar to experimental observations reported previously^5,40^. A possible explanation may be a constriction of the blood vessels and changes in blood vessel shape, as noted before^4^, where a change in vessel size was observed following laser irradiation. Since arterioles feed into vessels with smaller diameters as opposed to venules that feed into vessels with larger diameters, arterioles have a higher chance of coagulum interacting with the vessel wall downstream, resulting in flow stoppage. As observed in the FEM, shearing of the coagulum near the vessel wall is a likely failure mechanism in the coagulation process. Experimentally, shearing of the coagulum (Fig. 3, Panel C, blue arrow) near the wall was observed in cases where laser-induced hemostasis in larger blood vessels failed.

Coagulation of smaller-sized blood vessels followed expectations of the theory of selective photothermolysis, resulting in consistent stoppage of blood flow in vessels smaller than 100–200 µm (depending on blood vessel location with respect to the laser irradiation spot). Reports from other studies also suggest the inefficacy of pulsed dye lasers for coagulation of larger sized blood vessels and utilization of longer pulse durations to achieve better hemostasis^35–37,41,42^.

### Blood velocity and viscosity limitations

A limitation for laser photothermolysis is the relative speed of blood in larger vessels and associated higher shear stresses. Larger sized blood vessels (200/400 µm) were measured to support blood flow velocities of^43^ 20/40 mm/s. From the principle of selective photothermolysis, long pulse durations are recommended for blood vessels with sizes greater than^40,44^ 200 µm. For larger blood vessels, the profile of laminar blood flow is parabolic, with a 40 mm/s average flow velocity (corresponding to a transit time of 1 mm per 25 ms). This simple calculation suggests that the pulse duration recommendation derived from photothermolysis can fail within the transit time of convective heat transfer via blood flow.

The Nusselt number provides a measure of the ratio of convective to conductive heat transfer and varies from 0.2 to 1.2 for CAM vessels ranging in size^45,46^ from 0.05 mm to 1 mm. Interestingly, the initial temperature increase following absorption of pulsed laser light causes a viscosity decrease^33^ along with a density reduction (coagulum density being lower in solidified form due to expansion of blood cells forming spheroids as observed before^47,48^). Additionally, a finite time was reported for the formation of spheroids (through transient time in the formation of met-Hb as described before^47,48^).

### Limitations due to shear stresses near the vessel wall

Typical blood flow velocity profiles in a vessel lumen are parabolic, implying low shear stress near the vessel center, in contrast to the vessel wall where velocity gradients are steepest. Shear stress in large vessels is an order of magnitude (at least 10 times) higher than that in the microvasculature^43^ (< 100 microns) and can supersede the bonding energies coupling the coagulum with surrounding sites of the coagulation/vessel wall. Figure 3 shows two side-by-side cases of successful hemostasis vs unsuccessful hemostasis, where shearing near the wall (Panel C, Fig. 3) was observed, resulting in flow restoration (Panel D).

### Limitations from explosive bubble formation and rupture of blood vessels

Competing requirements for laser coagulation of large blood vessels are recognized. On the one hand, too slow heating may result in transit of the coagulum through the irradiation site before bonding with the vessel wall to complete hemostasis. Considering the exponentially decreasing absorption across the blood vessel cross-section, fluence distribution can result in the formation of vapor bubbles at the top of the blood vessel (toward incident laser irradiation)^25^.

### Results from proposed pulse stacking for coagulation in larger blood vessels

**Figure 4:**
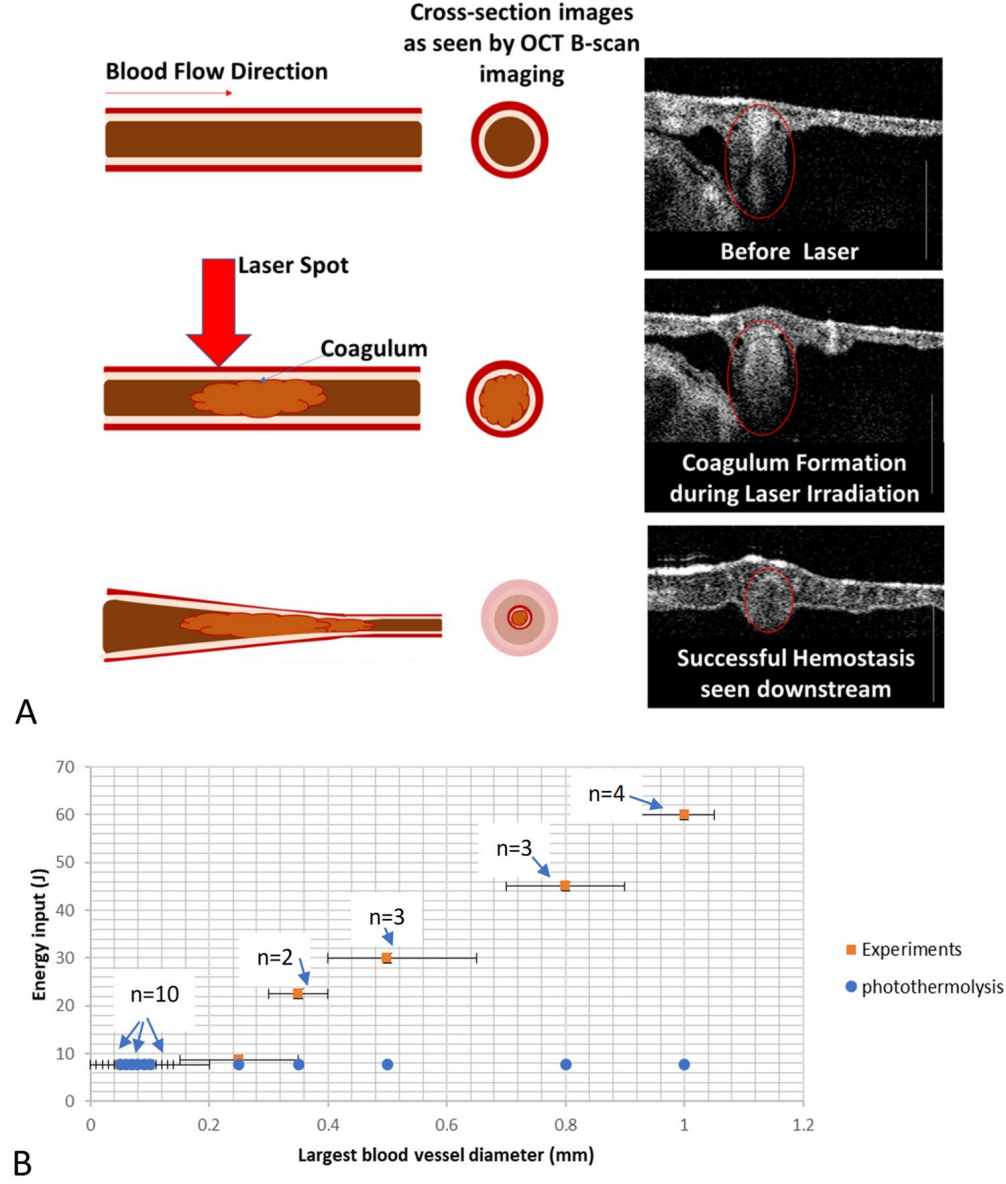
(**A**) Cross-sectional OCT monitoring of laser (1.07 µm) irradiation of CAM blood vessels. Downstream imaging across a blood vessel shows initial formation of the coagulum during laser irradiation, and after successful hemostasis, the blood vessel collapses downstream with no observable flow. Scale bar is 500 µm. (**B**) Radiant pulse energy (1.07 µm) determination with increasing blood vessel diameter in CAM arterioles for a 3 mm spot size. The number of trials at each vessel diameter is highlighted in the image with an ‘n’ number of trials/eggs. Blue circles indicate pulse energy computed from the theory of selective pulsed photothermolysis.

Initially, in response to laser irradiation, coagulum formation is observed growing from the center to edges of the vessel lumen (Fig. 4, middle panel). In some cases, where blood flow velocity (assumed to be dependent on the size of the blood vessel) was very high (> 50 mm/s), formation of a different-colored coagulum was observed (possibly met-hemoglobin^47,48^) that was subsequently displaced from the laser irradiation site by incoming cooler blood. Successful coagulation was observed with the enhanced stacked pulse scheme with a long duration initial pulse (in some cases approximately 5 times longer than that derived from the theory of selective photothermolysis). In some experiments, a rapid hemodynamic response was the result of successful hemostasis (Fig. S13), causing intense motions in the embryo post irradiation. OCT images highlighting hemostasis (Fig. S11) and brightfield photographs (Fig. S6) showed some hemorrhaging of capillaries around the outer rim of the laser irradiation zone. In cases where this initial energy titration was too high, explosive rupture/hemorrhage of the blood vessel was observed. Experimentally, laser dosimetry required for coagulation was computed for each vasculature. By carefully increasing the energy, coagulation success (Fig. 4, other examples in supplementary Figs. S11-13) was confirmed by histogram analysis of the resultant remnant vasculature.

FEM simulations provide a measure of the rate radiant energy that can be input into the vessel before causing explosive bubble formation within the blood vessel lumen and whether peak radiant power needs adjustment. Experimentally, irradiation times of 100–200 ms worked well for blood vessels in the range of 0.5–1 mm. Given differences in CAM vasculature in comparison to most tissues, where the CAM venules (furthest downstream from the heart) carry oxygenated blood and the arterioles (closest upstream to the heart) have deoxygenated blood, Hb has a higher absorption of 1.07 µm wavelength radiation, and the flow direction of venules (smaller into larger cross sections) makes coagulating CAM venules more challenging compared to CAM arterioles (flow into smaller cross-sections increasing likelihood of coagulum sticking to the wall or stopping flow). The CAM scenario is reversed in most tissues where the oxygenation Hb/HbO_2_ content in venules/arterioles is juxtaposed. Additionally, given the arterial structure (possessing thicker and more elastic walls than venules), explosive rupture was more commonly observed in initial laser coagulation trials within CAM venules than in CAM arterioles. In addition, coagulation of CAM venules was less successful than CAM arterioles (Fig. 3). These results are consistent with the fact that CAM arterioles were more uniformly heated given the higher penetration depth of 1.07 µm light in deoxygenated blood, and CAM venules experienced increased temperatures toward the incoming radiation direction, causing localized cavitation and rupture (flow in CAM venules was faster than in arterioles^32,43^). To prevent local cavitation and vessel rupture, the peak power for laser irradiation into CAM venules was reduced (halved) so that to maintain constant energy input, the laser irradiation times of the pulse sequence were proportionally increased (doubled). Frequency of cavitation and vessel rupture was reduced, and more successful coagulation was observed in CAM venules after reducing the peak laser power while keeping the radiant energy fixed.

In addition to major factors, including visco-velocity profiles and shear stress, another source of discrepancy between photocoagulation of smaller and larger blood vessels may be the relative size between the beam spot and blood vessel diameters. Differences in CAM vascular networks of an arteriole versus venule: As discussed earlier, although optical absorption in arterial vs venous blood is different (venules appear bright red upon visual inspection and carry oxygenated blood in CAMs, while arterioles carry deoxy hemoglobin, which is juxtaposed in most tissues^43^), arterials have a higher likelihood of coagulation than venules, as reported in previous CAM studies^40,49^ using 585 nm irradiation. In the CAM vasculature, blood vessels develop in artery/vein pairs^43^, as observed in experiments reported above (Fig. 3, corresponding enface Fig. S10). In this case, the suspected arteriole underwent successful hemostasis, while blood flow was observed in the venule post irradiation. Beam Size Limitations: Compared to capillaries and smaller blood vessels, the length to volume ratio exposed to laser irradiation is significantly different for larger blood vessels. Time duration of Arrhenius damage: Arrhenius damage threshold temperatures and corresponding time durations vary in blood vessels compared to surrounding tissue and the collagenous arterial vessel wall^41^. This suggests that the time variation of temperature for achieving hemostasis has a complex dependence on these factors given that the condition for success is Arrhenius damage in these tissues (blood, arterial collagen). Successful coagulation of vessels larger than 150 µm is challenging when laser dosimetry is derived from the theory of selective photothermolysis^37,41,42^.

Laser coagulation of blood vessels with large cross-sections and corresponding high blood flow rates was investigated in this study. A near infrared fiber laser (1.07 µm) was utilized to investigate blood vessel coagulation. The existing literature reports numerous studies of blood vessel coagulation in the treatment of pediatric vascular lesions, such as port-wine stains for microvascular hemostasis using pulse dye lasers. In addition, infrared lasers have also been successfully utilized in the long pulse regime for performing hemostasis equally well or better than pulsed dye lasers^34–36,41,42^. Laser (1.07 µm) dosimetry for coagulation of large diameter vessels with high flow rates was investigated using the CAM vascular model and highlights the limitations of our current understanding. Limitations of the theory of selective photothermolysis for hemostasis of large blood vessels with high flow rates were investigated, suggesting a hypothesis that current limitations originate from high shear stress variations in large blood vessels along with temperature-dependent viscosity and velocity changes. FEMs that include fluid flow and thermal diffusion were developed that aid in contextualizing and testing limitations in hemostasis resulting from laser irradiation. Fluence rate parameters were then adjusted (not only accounting for some of the recommendations^25,44^ involving Monte Carlo simulations to avoid explosive vaporization pockets during irradiation) based on experimental observations utilizing OCT angiography (OCTA), resulting in consistent coagulation.

Future work that may improve the methodology is to study coagulum formation and develop an FEM model with a frontier tracking methodology^50^. A visual inspection of the coagulation site during laser irradiation shows an accumulation of the coagulum in the direction of the flow. This effect may be due to the reduced density making the coagulum flow faster relative to surrounding uncoagulated blood causing the frontier of coagulum formation to have a skewed shape in the flow direction, which could be simulated with some of the frontier tracking methods introduced and discussed by Tryggvason et al. Additionally, considering the asymmetry between the leading and lagging fronts of the coagulum and changes in visco-velocity profiles, in future work, a two-beam approach where the leading edge of the blood flow experiences higher power photocoagulation irradiation whereas the lagging edge is irradiated with subthreshold shear velocity modifying radiation may help improve photocoagulation and achieve successful and consistent hemostasis. Additionally, this could be achieved by applying mechanical pressure to reduce the diameter of the vessel, similar to current bipolar clamping devices, to coagulate vessels in the 2–8 mm diameter range^39,51,52^. Another limitation in coagulation of blood vessels with an incident collimated beam is the concentration of fluence on the side closer to the irradiation, sometimes causing explosive cavitation resulting in rupture of large blood vessels. This limits the average power input into blood vessels. A dynamic focusing methodology may be explored to focus the light according to a depth-resolved OCT image to cause uniform heating accounting for scattering properties at NIR wavelengths, thus improving coagulation success for larger sized (2–5 mm) blood vessels where currently clamping devices have been utilized^51,52^. To model these effects, a frontier tracking methodology that has been utilized in predicting the performance of molten metal solidification^50^ can provide additional insight into the hemostasis process. This methodology hypothesized and successfully tested the effects of the velocity profile along the solidification phase front along with the effects of shear stress. An FEM incorporating a frontier tracking approach along with the viscosity and flow dynamics incorporated in this report can provide a more accurate description of the coagulation/hemostasis process. Another potential limitation of the FEM model is incorporation of viscosity dependence utilized as a direct relationship between viscosity and temperature directly derived from published experimental results^33^. The correlation between temperature and viscosity (Fig. 1B,C) is observed at the vessel wall with reduction in viscosity and temperature due to thermal diffusion. In reality, the viscosity may reach a steady state value and a frontier tracking methodology^50^ may aid in incorporating any potential hysteresis involved in the relationship of viscosity with temperature. Additionally, another potential limitation is that a flattop beam was simulated versus non-flat top (gaussian-like) beam intensity profile utilized in experiments. Finally, given that cautery tools are able to coagulate up to 7 mm diameter blood vessels, further experiments are required to study the possibility of laser coagulation of blood vessels (e.g., up to 7 mm diameter). Finally, if the laser induced coagulum were to fail, the coagulated blood in larger diameter blood vessels (500 µm) could result in complications such as an embolism. Future studies are needed in larger blood vessels (> 3 mm) potentially using a larger animal like a rabbit femoral model.

## Materials and methods

The theory of selective photothermolysis, in the context of blood vessel coagulation, suggests that pulses of light with a wavelength targeted for absorption by hemoglobin with appropriate pulse duration and fluence can heat blood vessels to achieve coagulation and hemostasis without causing nonspecific damage to surrounding tissues^1,6,53^. In the theory of selective photothermolysis, the duration of pulsed laser exposure is governed by the thermal relaxation time of the target vessel. For vascular coagulation, the blood vessel thermal relaxation time varies with the square of the lumen diameter (*D*), Eq. (1) prescribes a quadratic relationship between vessel thermal relaxation time and blood vessel diameter with an inverse relationship to thermal diffusivity. From prior art^54–56^, a typical value for thermal diffusivity (α) is 0.15 mm^2^/s in relation to Eq. (﻿1). Vessels of larger size (e.g., millimeter sized) diffuse heat slower into surrounding tissues compared to smaller sized structures. In the theory of pulsed photothermolysis, convective heat transfer due to blood flow through the laser irradiation spot is ignored. This assumption is justified by the significantly shorter laser pulse duration compared to the duration of blood flow through the irradiation spot—the blood is viewed as stationary during laser irradiation. Constraint on laser pulse duration in Eq. (1) is designed to ensure substantial confinement of deposited heat during irradiation.

The temperature increase to coagulate target blood vessels is governed by time–temperature first-order rate kinetics given by the Arrhenius thermal damage integral^25,44,57^. The temperature increase (*ΔT*) is governed by the absorption coefficient (after accounting for the fluence distribution by scattering and absorption to surrounding tissue regions^58,59^, Eq. (2). The temperature increase (*ΔT*) from the absorption of pulsed laser radiation can be computed using the absorption coefficient in blood (*µ*_*a*_) as given in Eq. (2).

1 $$Thermal \,relaxation\, time\, (s)=\frac{{D}^{2}}{16\alpha }$$

2 $$\Delta T = \mu_{a} \frac{\Phi }{\rho C}$$

Here, µ_a_ corresponds to the absorption coefficient of blood at 1.07 µm (approximately 0.3 mm^−1^), and fluence (Φ, J/cm^2^) is computed for a given laser spot size (e.g., 3 mm here). *ρ* and* C* are the density and specific heat (product yields 0.004 J/mm^3^/K). Yb fiber laser pulse durations and peak powers were constrained to limit the increase in blood temperature to greater than 55 °C. The peak power limit on the Yb fiber-laser module utilized in the study (see supplementary 2.1) was 3000 W (10% max. duty cycle, 300 W Avg.), the pulse duration was fixed at 50 µs, and the repetition rate was adjusted to account for the 10% duty cycle limitation (2000 Hz). If a large vessel receives too much radiant energy (to cause local temperature T > 100C) over a short pulse duration, the blood vessel can rupture, as water in the top of the vessel can create a vapor bubble and possibly burst the vessel wall, resulting in hemorrhage. Conversely, if a small diameter blood vessel absorbs radiant energy through a low power pulse over a longer period of time, the vessel will not coagulate as heat will have diffused out of the lumen without hemostasis. In the theory of selective photothermolysis, the required laser dosimetry is determined by the requisite pulse energy and thermal relaxation time (Fig. S4).

An FEM was developed to investigate the observed limitations when coagulating large diameter blood vessels. COMSOL FEM simulation software allows for concurrent mass and heat transfer simulations in materials encompassing these domains. A multiphysics model consisting of individual modules for heat transfer in liquids and mass transfer is proposed to investigate limitations observed in experiments targeting large diameter blood vessels for laser hemostasis.

To investigate the temperature distribution and fluid transfer of light-generated heat in vessels with fast-flowing blood, a 3D FEM was constructed. A COMSOL heat transfer module was utilized to compute the thermal relaxation in a cylindrical blood vessel, with the surrounding media being tissue. The COMSOL Multiphysics simulation incorporated heat transfer (ht) in fluids, flow (spf) and the bioheat transfer model (ht2). Multiphysics simulations were run in transient mode in a 3D coordinate system. Flow was incorporated into the model via the fluid laminar flow module linked with the heat transfer module via COMSOL Multiphysics. The peak value of the parabolic flow distribution was derived from experimentally measured flows (e.g., approx. 40 mm/s peak velocity for 400 µm blood vessels)^43^. In addition, temperature-dependent viscosity was modeled by parametrically changing the temperature from data reported previously^33^. Input to the 3D FEM was absorbed fluence determined from Monte Carlo simulations (see supplementary Sect. 3). Second, to reduce the computation time for a 3D model, a symmetric 2D FEM in COMSOL was used. 3D model incorporating blood flow and thermal diffusion with optical fluence input resulted in long simulation times (> hours) to converge whereas a 2D FEM model allowed for simulation in a few minutes allowing for multiple time scale iterations. Different sized blood vessels and their corresponding parabolic velocity profiles were input into the model from prior reports^32,43^. For the flow (spf) simulation, dynamic viscosity of blood was allowed to vary with temperature based on experimentally obtained viscosity values^33^. According to the experiment and considered model in this study, the dynamic viscosity reduces up to 55 °C before increasing as determined through rheology measurements reported previously^33^. An Arrhenius kinetic model of protein coagulation was added to the model to include the respective parameters corresponding to blood inside the lumen and tissue outside the lumen.

OCT angiography images were recorded of CAM blood vessels in conjunction with irradiation by a Yb fiber laser (1070 nm) coaligned bench-top system (see supplementary Sect. 2). A histogram of CAM blood vessel diameters was constructed from recorded angiography images to derive a laser irradiation protocol (see supplementary Sect. 2.2) for the CAM vasculature. Additionally, OCT images were recorded during laser coagulation of larger CAM blood vessels in response to the proposed enhanced stacked pulse irradiation scheme. In all CAMs that were imaged and irradiated (n = 15), pre- and post- irradiation images were recorded with OCT angiography. All the irradiation experiments were carried out at approximately day 8 of the gestation period and all methods were carried out in accordance with relevant guidelines and regulations (please see supplementary methods section for detailed overview of the CAM experiment protocols).

## Supplementary Information

Supplementary Video 1.

Supplementary Video 2.

Supplementary Video 3.

Supplementary Information.
