# Supplementary figures and images for: Laser coagulation and hemostasis of large diameter blood vessels: effect of shear stress and flow velocity
Source: Sci Rep. 2022 May 19;12:8375. doi: 10.1038/s41598-022-12128-1 (PMC9120470; doi:10.1038/s41598-022-12128-1)

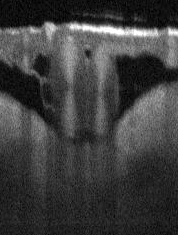

Supplement: Supplementary file 3 — Supplementary Video 3. [file 41598_2022_12128_MOESM3_ESM.gif]
