# Supplementary material for: Laser coagulation and hemostasis of large diameter blood vessels: effect of shear stress and flow velocity
Source: Sci Rep. 2022 May 19;12:8375. doi: 10.1038/s41598-022-12128-1 (PMC9120470; doi:10.1038/s41598-022-12128-1)
Supplement: Supplementary file 4 — Supplementary Information. [file 41598_2022_12128_MOESM4_ESM.docx]

Supplementary Information Text

1. **Protocol for CAM incubation:**

A standard chicken egg incubator was first cleaned with water and then disinfectant to ensure no contaminants or mold inside it ^1,2^. Fertilized eggs (AgriLife-Poultry Science, Texas A&M University) were then purchased and placed inside with the wide side up. The day these eggs are kept inside the incubator marked start of CAM age (counting in days). From day 1, since the eggs were placed inside the incubator, the humidity and temperature were monitored by appropriate apparatus and maintained at 37^o^C and 40-60% humidity (by placing distilled water in tracks of incubator accordingly). Every day until day-3, the eggs were turned at least twice a day by tilting 45 degrees to the right, and then 45 degrees to the left. On day-3, eggs were placed under a flashlight to monitor the yolk, under the light holes were drilled while avoiding breakage in yolk portion (Fig. S5). Approximately 4mL of albumin was removed via a sterile syringe. Like in case of drilling, syringe was maneuvered to avoid yolk area. Liquid extracted should be almost entirely transparent. One day 4, surgical scissors were used to cut about 20mm diameter holes on top of the eggs. Once this was successfully done, sterile petri dishes were used to cover the eggs before replacing them in the incubator, care was taken not entirely block air into the top. On subsequent days, humidity and temperature were monitored to maintain at 37 C and 40-60% humidity. Also, eggs that were non-vascular were removed in order to avoid complications of infection to the other eggs. From day-7 significant vasculature was observed in eggs that incubated successfully. Albumin levels inside the eggs were noticed to be lower with progression of days in CAM age. From day-8, experiments may be carried out for coagulation. Every day, growth can be observed in hematicrit concentration and blood vessel size ^3–5^

1. **Bench-top system for acquisition of blood vessels information**

A bench-top bulk applicator is described here to study the effect of fluence irradiations obtained depending on the vessel distribution and monitored pre/post laser irradiation. The bench-top applicator had two major subsystems.

- 1. **1070 nm Fiber laser:**

A Yt fiber laser (IPG Photonics) configurable for either QCW (Quasi CW) or CW operation was used for coagulation/hemostasis. For coagulation, the laser was operated in QCW mode (50µs to 200ms programmable duration) with variable average power (max 300W). The fiber core size of the laser was 50 µm with an NA of 0.09. The light emitted from the fiber was collimated (IPG Photonics Head) and directed onto a di-chroic mirror (DM, DMSP1180, Thorlabs) to combine Yt and OCT beams. Collimated beam size was measured with a knife edge to about 3mm.

- 1. **OCT imaging:**

The OCT system utilized a swept-source, mode-locked laser (Axsun, Billerica MA) with emission at 1310+70 nm and a scan-rate of 100kHz. Laser output was coupled into a fiber-optic (SMF-28) Mach-Zehnder interferometer with pathlength and dispersion matched sample and reference paths. Sample path light was directed onto two galvanometer mirrors positioned in a telecentric configuration (Fig. S6) with an aspheric ZnSe scanning lens (AR112-ZC-XWL-25-50, ISP Optics). Light backscattered from the sample and reflected from the reference mirror interfered and was directed onto balanced detectors (BD). Recorded tomograms (512 × 512 × 736 voxels) had a typical field of view (FOV) of 10 × 10 × 5 mm^3^ with a longitudinal spatial resolution (depth dimension) of 6.75 μm (in air).

- - 1. **Angiography from OCT imaging**

OCT angiography techniques detect the temporal shift in red blood cell (RBC) position and derive volumetric flow images from decorrelation of analytic signal interferograms (borrowed from supplementary section of our previous work^6^). OCT-based angiography approaches quantify an average decorrelation observed within a depth window of an A-scan over a number of repetitions ^7–14^. The angiography computation was implemented on a GPU (RX-480P8DFA6 AMD), to obtain an angiogram within 90 seconds of acquisition using the following equation.


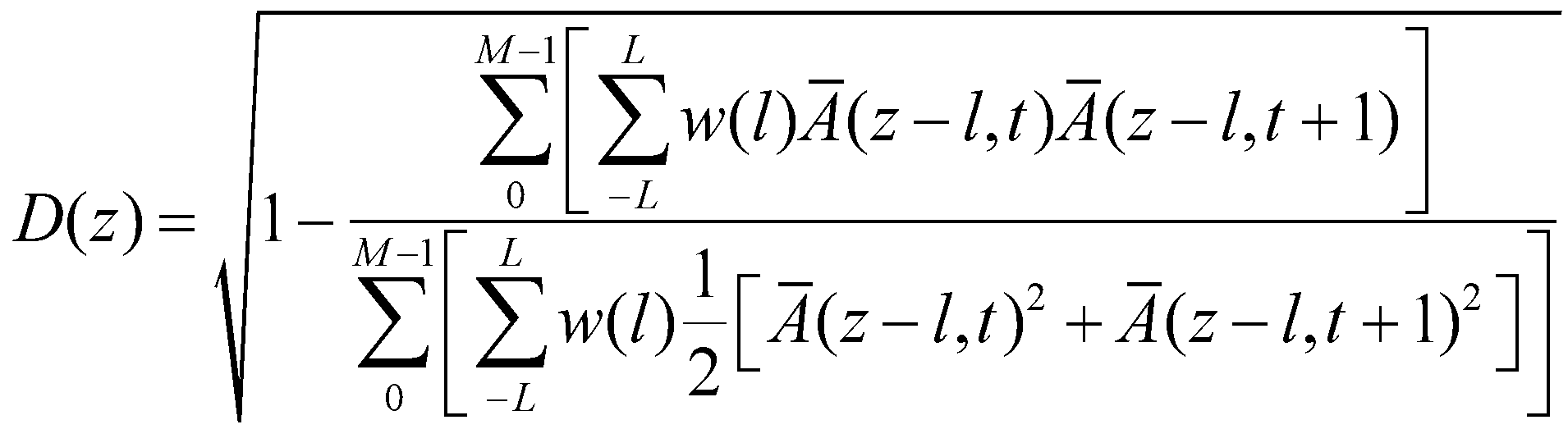


Eq. 3

- - 1. **Histogram analysis for obtaining size distribution of blood vessels**

The vessel diameters distribution was estimated with a semi-automatic matlab script, where the operator highlighted two regions across a blood vessel to obtain the blood vessel size (in pixels). For about half of the cases, a selected number of data points were input after a pre-coagulation angiography step to computer a histogram of the distribution of blood vessels. For these blood vessel sizes, a look up table computed the appropriate pulse energy/peak power, pulse durations for the subsequent laser irradiation.

1. **Photo-thermolysis:**
   1. **Differences in absorption coefficient in CAMs with age (day)**

In 1971, Hiroshi Tazawa ^5^ measured the change in different parameters in relation to the vasculature of CAMs. These parameters include Hct (Hematocrit %), radiometer blood gas instrument for O_2_ pressure CO_2_ pressure in addition to O_2_ capacity/saturation through the different stages of embryonic development. This in relation to optical property characterization dependence on Hct presence highlights the importance of age of the egg while adjusting the dosimetry of the laser for achieving coagulation ^15,16^. The value for µ_a_ for blood in CAMs, in comparison to computations with human blood properties ^15,16^ can be underestimated due to at least two factors: 1) the blood is more deoxygenated than oxygenated in comparison to typical blood oxygenation ^15,16^; 2) the hematocrit of the blood may be less that the 42% assumed depending on the age of the CAM. For example, hematocrit on days 8 and 9 are at about 22% ^5^ which would reduce the absorption by about half in comparison the calculations obtained for human blood, implying the value used in the computations/simulations are two times larger, meaning the powers required will be about 2x larger compensating for this absorption coefficient. Given, the average power limitations of the 1070nm laser in this work, one possible avenue for consideration may be applying multiple 300W pulses to achieve hemostasis. This is explored in the following sections.

- 1. **Monte-Carlo simulations for laser coagulation**

From the thermolysis theory, a limitation in the peak power that can be observed in the above description is the fluence rate recommendations for blood vessels less than 60 µm. The peak power required to satisfy the fluence for successful coagulation exceeded the maximum peak power supported by the laser module (1.07um, 3000W peak power, 300W average power, IPG Photonics). To account for this, monte carlo simulations (Steven Jaques)^17^ were carried to obtain a pulse pattern that would satisfy the Arrhenius damage integral requirement. In addition to the quick thermal relaxation times, blood flow heat transport in neighboring regions provides for an enhanced relaxation through convection. Finally scattering in large blood vessels, given the geometry of the illumination with respect to blood flow direction, has been attributed to heat localization in regions closer to the irradiation zone ^18–21^. To simulate all these effects, finite difference methodology was proposed ^19,20,22^. These methodologies were incorporated to perform temperature finite difference and arrhenius computations in MATLAB. Detailed description of the FD methodology and monte carlo simulations can be found elsewhere ^19,20,22^. A summary of these results is presented here whose results were analogous to some of the existing literature observations ^19,21,23^.

Location of the blood vessel was varied in depth for case of large blood vessels given the attenuation of light experienced as well as size to study any effects of vessel placement in the field of the laser irradiation. Given the long penetration depth with limited scattering in CAM background tissue uniform irradiation was simulated in different size of blood vessels with varying depth of placement even with changing size upto 1mm. To further the simulation for incorporating complex vessel networks, a candidate vessel network reported in ^24^, was simulated with a collimated 1.07 µm irradiation. Peak power fluence rates was computed to be limited through the monte carlo simulations to provide a threshold above which vaporization temperatures are caused within the vessel towards the irradiation site. The fluence rates were limited by this maximum average energy.

For the case of a smaller vessels in the range of 50 µm, photo-thermolysis required relatively large peak powers. The irradiation peak power possible with current source was limited to 3000W. In order to come up with a pulse pattern for such a vessel on the surface of the tissue, a multiple pulse pattern was tested in monte carlo simulations coupled with finite difference analysis to compute the temperature gradient maps. It is noticed that that multiple irradiations are needed given the small energy deposition seen in smaller vessel to cause coagulation along with relatively fast relaxation times. After 5 pulses separated by 500µs each (quickest repetition rate possible with current laser in the study), a reasonable arrhenius threshold of damage is reached. Arrhenius damage is plotted in time, highlighting damage. The relaxation time is about 1ms close to the estimation from Eq.1, forcing a multiple requirement in order to reach a reasonable arrhenius damage. To explore the idea of localized damage, two locations away from the center, left and right at 1mm distances taken show the temperature increase is insufficient to cause any damage and relaxed to the nominal temperature of about 40^o^C. In both these cases, the Arrhenius resulted in low damage.


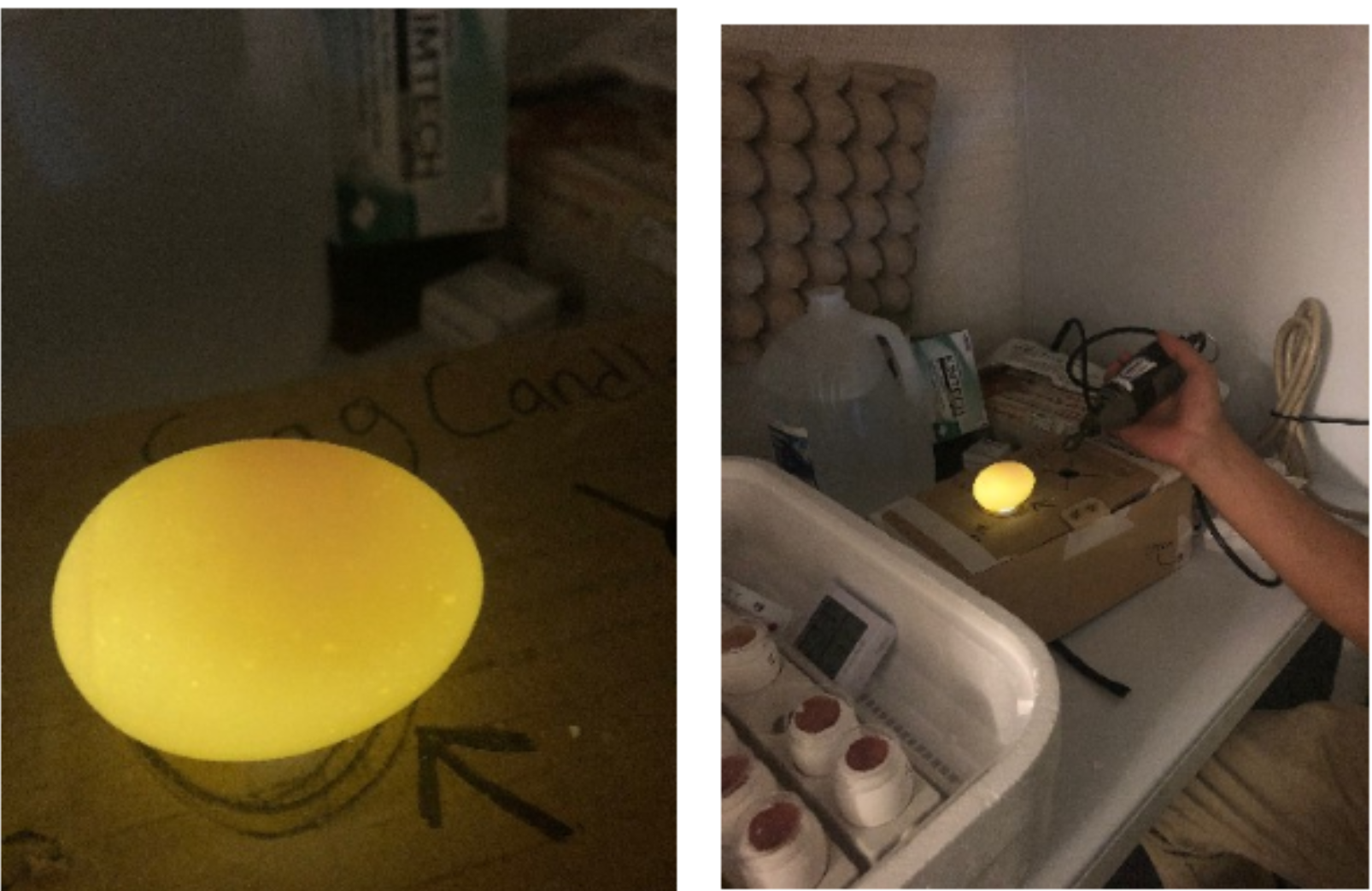

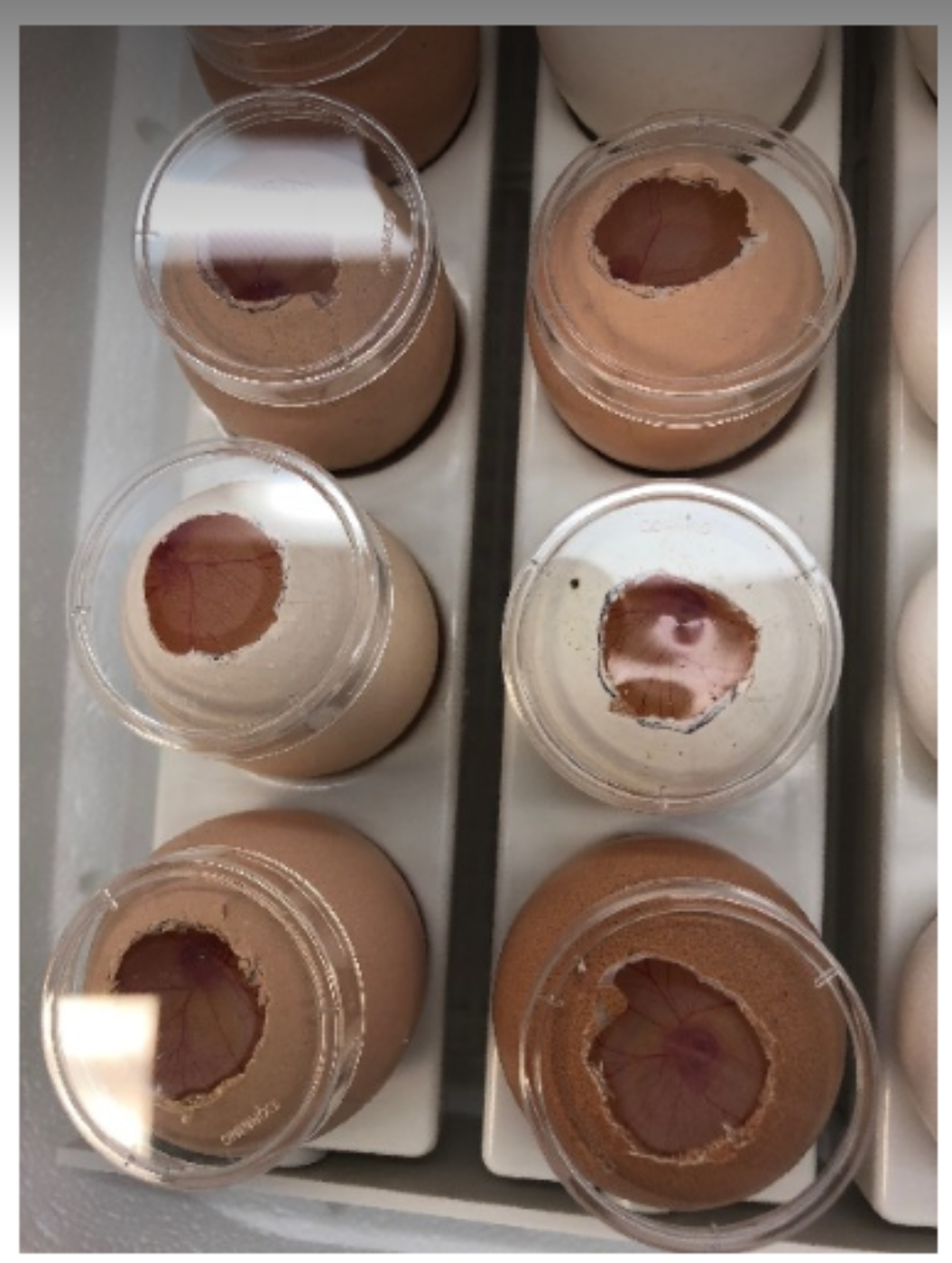


Fig. S1 CAM incubation stages described. Egg candler station (left) for locating the yolk in the egg. Drilling process (middle) highlighted through guidance of the egg candler. Post extraction and opening (right) of the eggs on Day 4 of the embryonic development state revealing vasculature


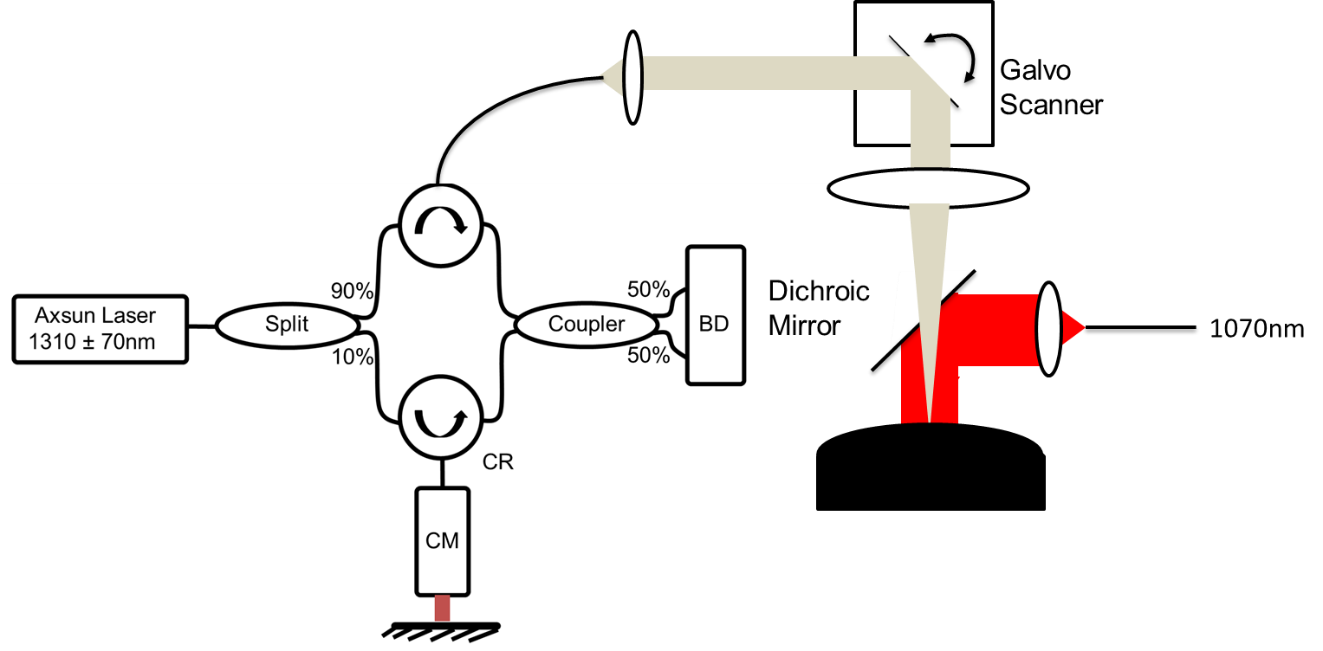


Fig. S2. Benchtop OCT co-aligned Yt laser surgery coagulation system. A Mach–Zehnder fiber Interferometer uses circulators (CR) and balanced detection (BD) and is dispersion compensated (CM). Yt and OCT beams are fiber delivered via collimators (RC) and combined with di-chroic mirrors (DM). The Yt 1070nm beam is collimated onto the sample, whereas the OCT beam is focused to a spot size of about 40 µm.


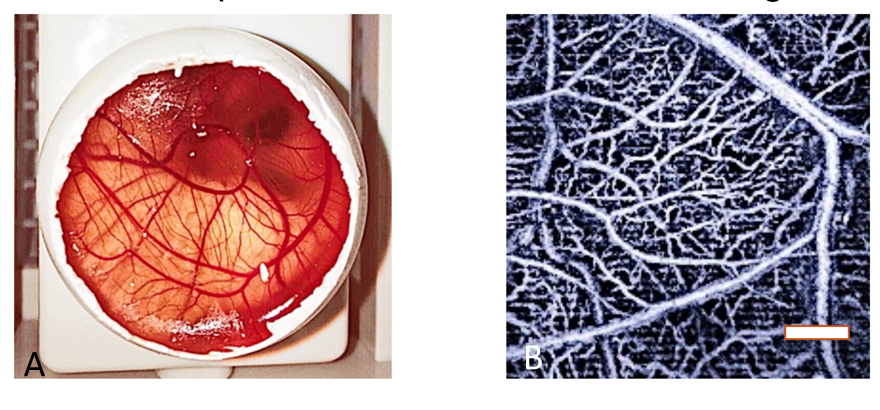


Fig. S3*.* A) Blood vessel formation after CAM incubation (Day 8) B) OCT angiography information of CAM vasculature. Scale bars 500 µm.

.


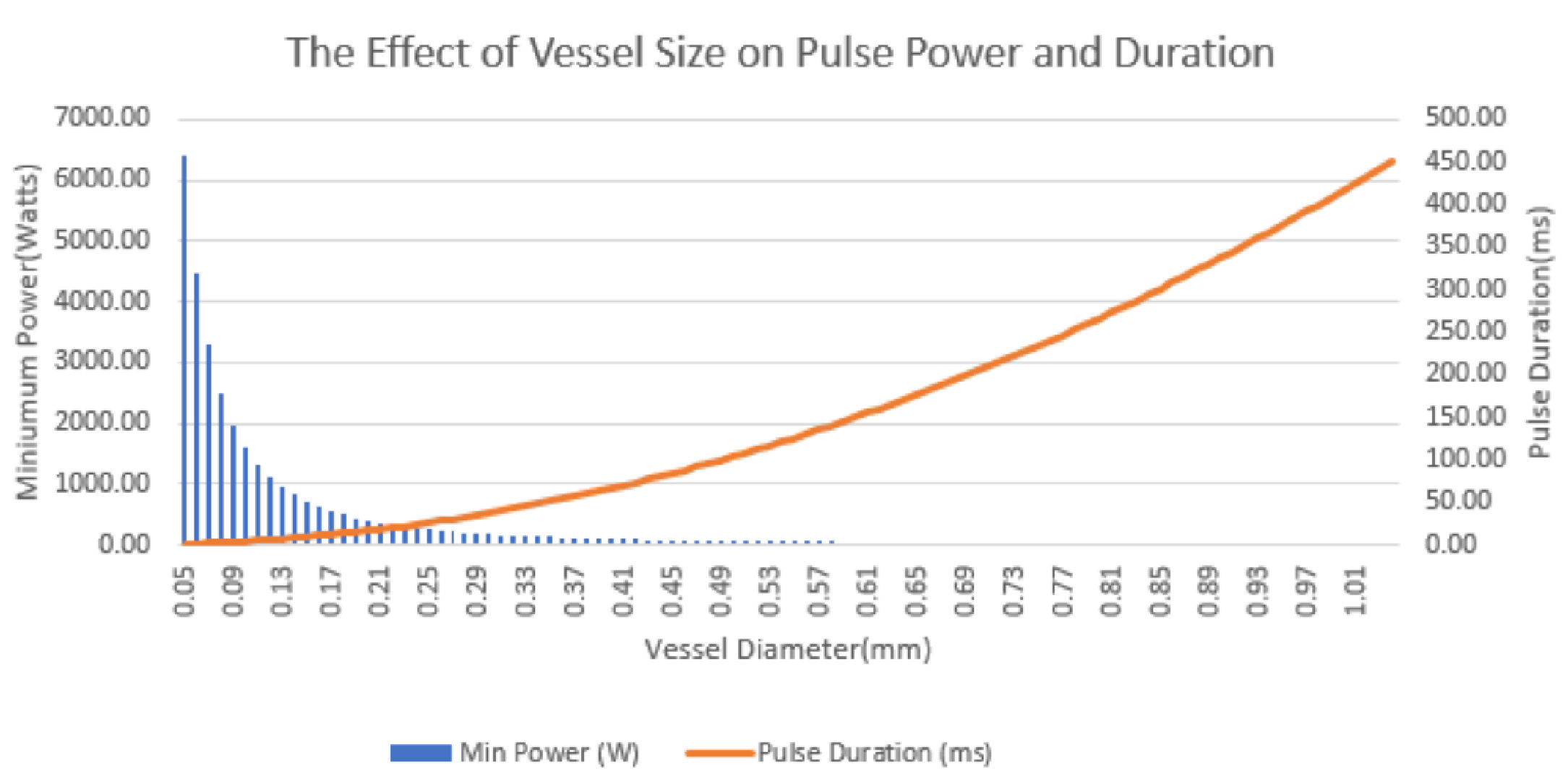


Fig. S4. Peak power required for photocoagulation of blood vessels of varying lumen diameters as derived from theory of selective photo-thermolysis. Coagulation of smaller blood vessels requires higher peak power so that heat does not diffuse into regions surrounding the vessel lumen, whereas large vessels can be heated with longer pulse durations and lower powers.


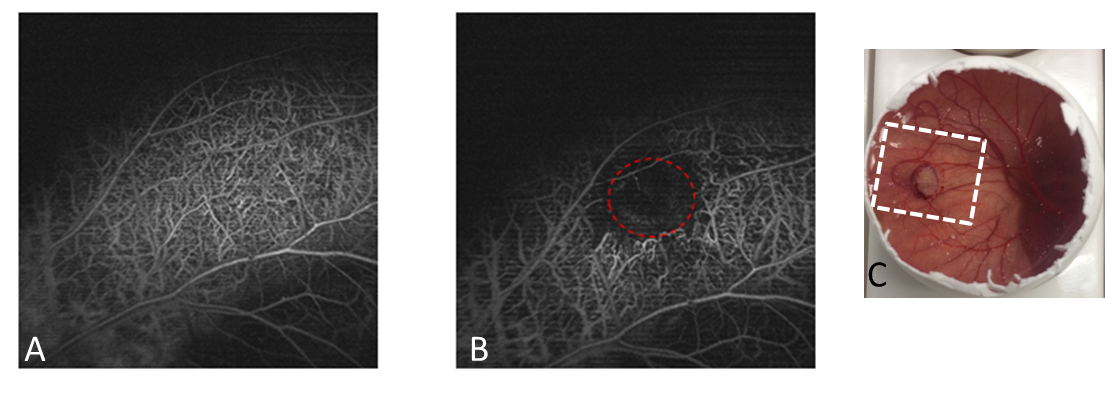


Fig. S5*.* A. Pre-coagulation OCT angiography image . B. Post-coagulation OCT angiography image. C. Visible image of CAM irradiation site (image field 10 x10mm2). Laser dosimetry: approx.10J, 25ms, Ф3mm.


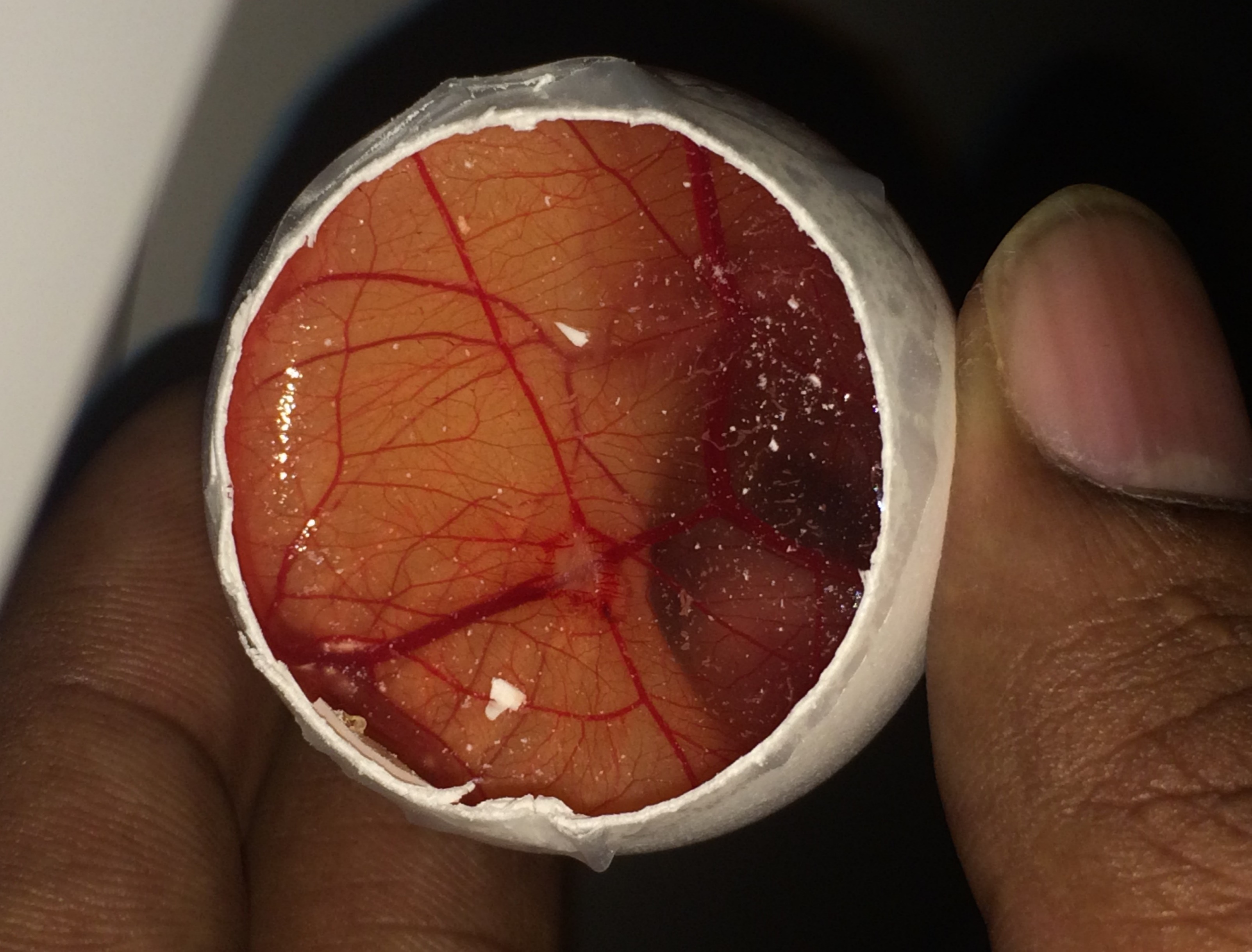

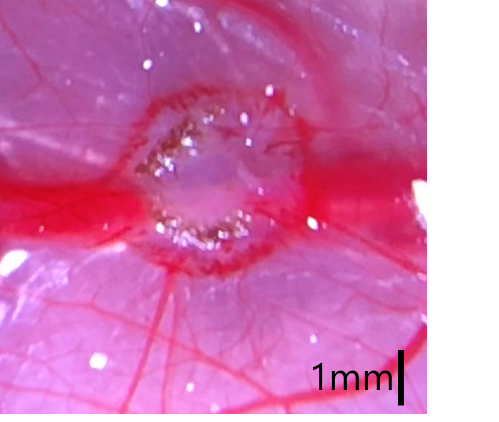


Fig. S6*.* Immediately post-coagulation brightfield photographs of CAM vasculature with a large vessel (image field 6x6 mm^2^). Left vessel was bright to visual inspection (indicating vein) and the large vessel in right image was darker (indicating artery). Minor hemorrhage is observed at the outer rim of the laser irradiation site.


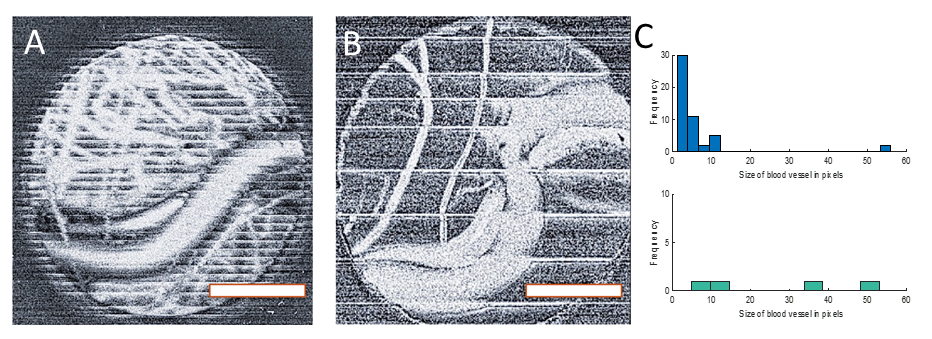


Fig. S7*.* Pre-coagulation angiography image from OCT (Panel A). Post-coagulation angiography information (Panel B). Scale bars are 500 µm. Histogram analysis of blood vessel size distribution showing hemostasis in all the smaller vessels (Panel C).


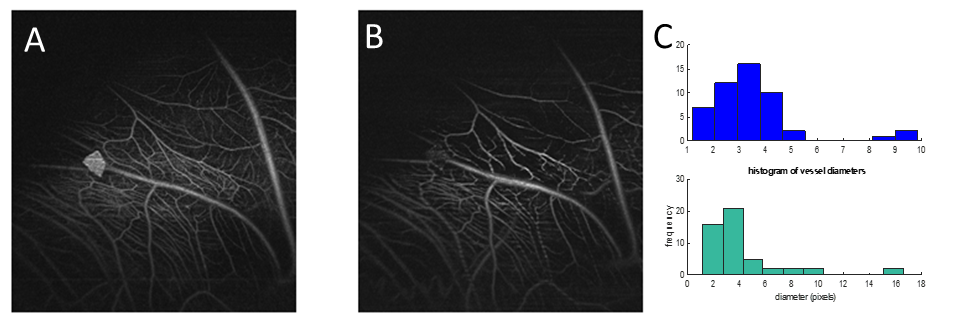


Fig. S8. Coagulation in another case of Egg vasculature with laser irradiation recommended. Again. Larger blood vessels had a lower likelihood of stopping compared to smaller microvasculature and capillaries. Image FOV is 10x10mm. Histogram analysis of blood vessel size distribution showing hemostasis only in smaller vessels (Panel C, blue histogram is blood vessel size distribution before laser irradiation and green is after.).


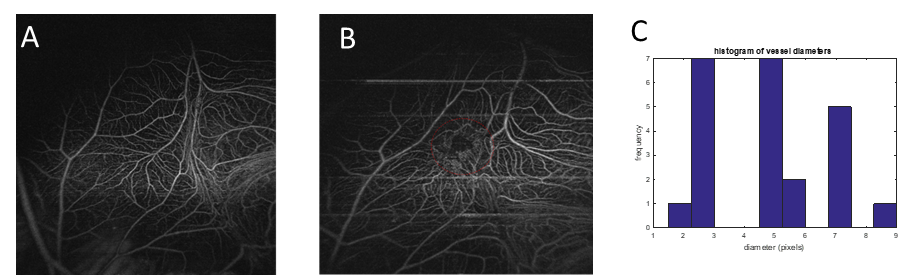


Fig. S9*.* Complete stoppage seen in vessel networks with mean size less than 200 µm. Image FOV is 20x20 mm.


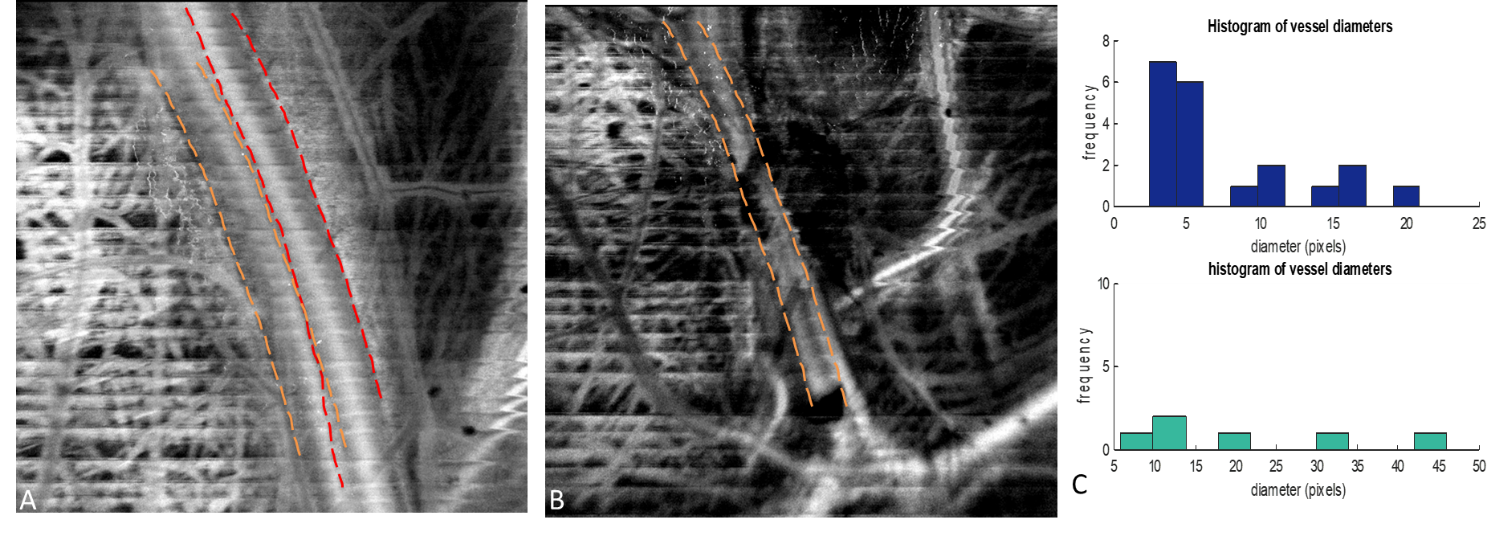


Fig. S10*.* arterial (red)/venule(orange) comparison for same irradiation. Arterial successfully coagulates, while venule is still intact. C) histogram analysis showing one larger vessel intact, while smaller vessel stop flowing. Image FOV for Panels A,B is 5x5 mm.


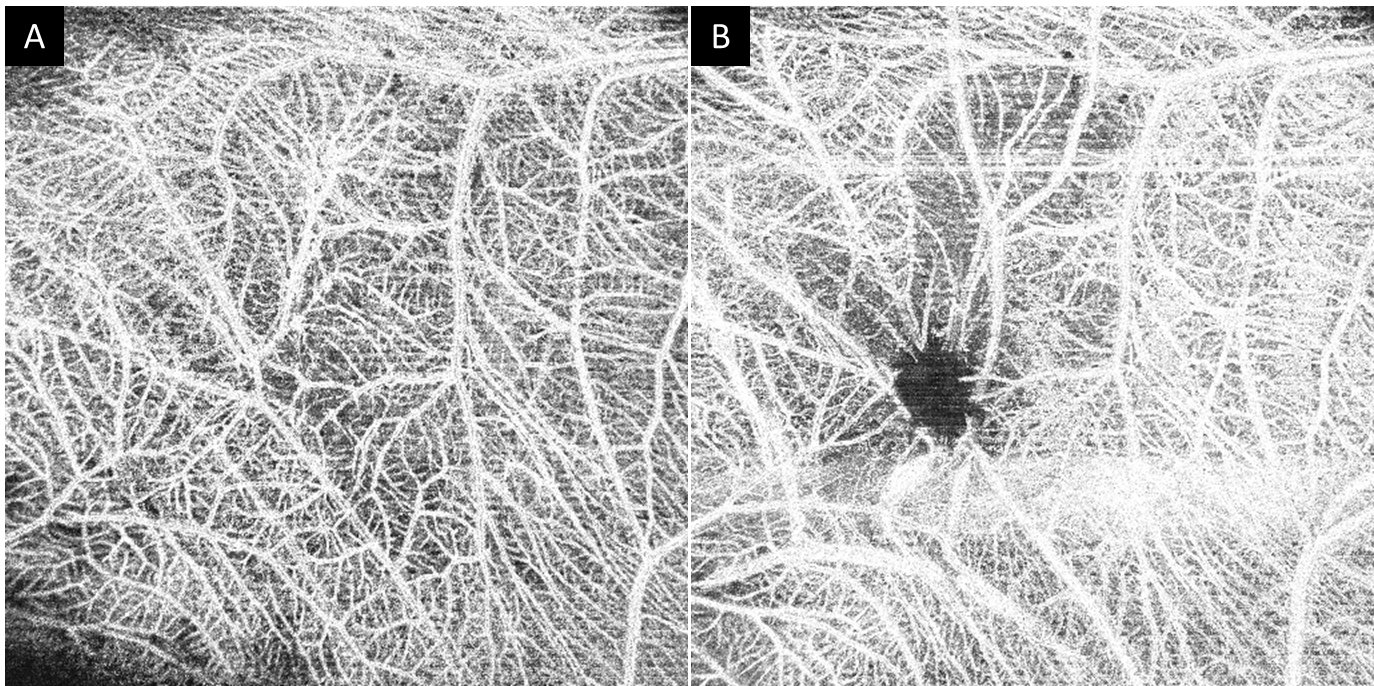


Fig. S11*.* Pre-coagulation angiography image from OCT (left, Panel A). Post-coagulation angiography information (right, Panel B) (image field 20x20mm)


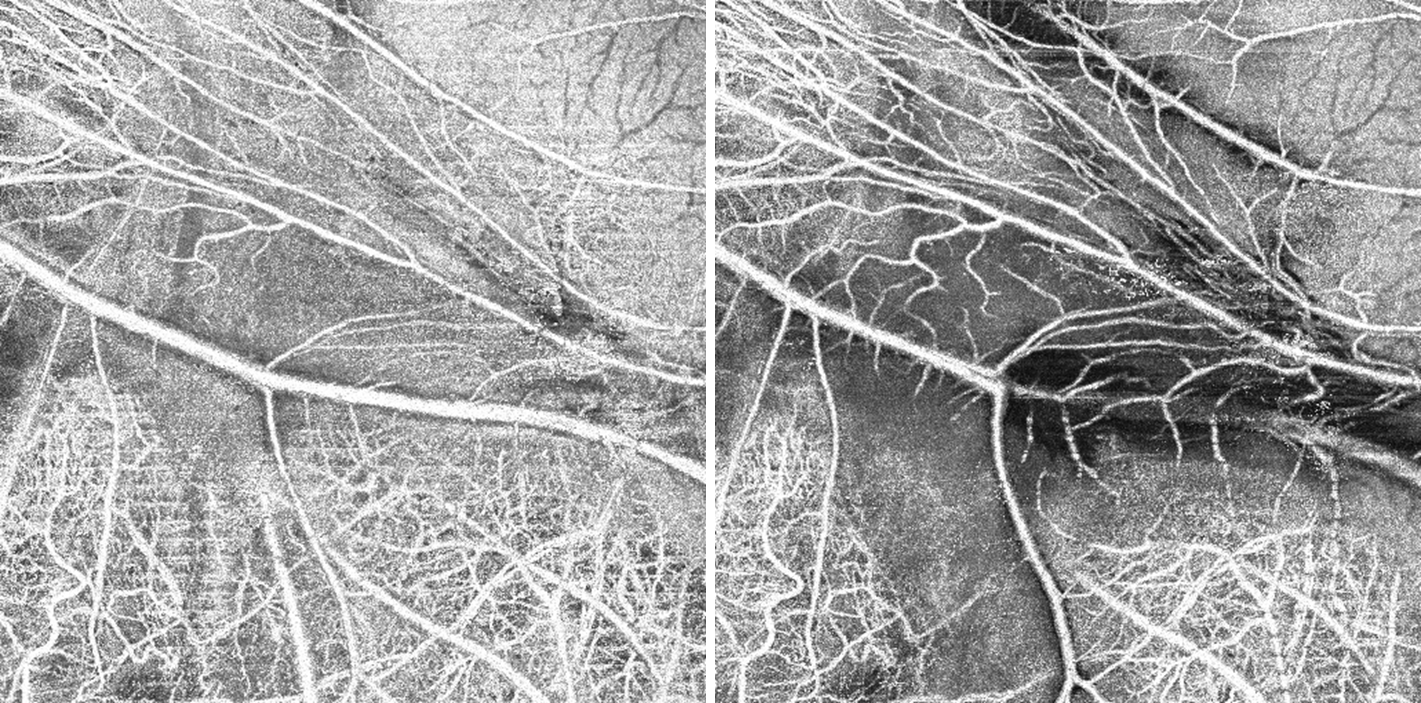


Fig. S12*.* Pre-coagulation angiography image from OCT (left, Panel A). Post-coagulation angiography information (right, Panel B) (image field 10x10mm)


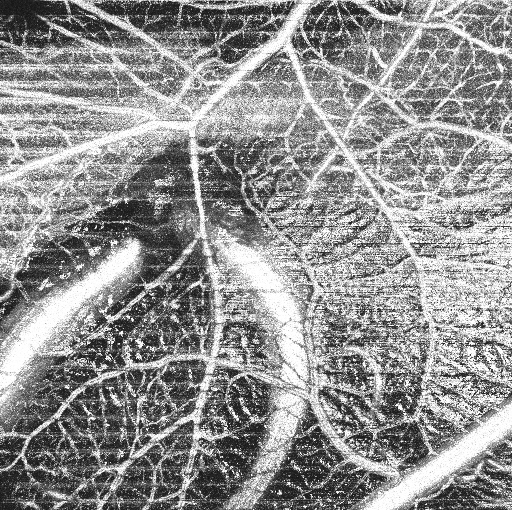

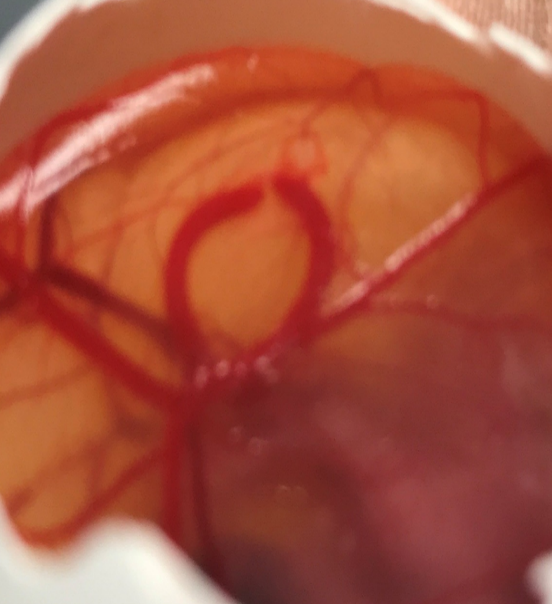


Fig. S13. Post-coagulation angiography image from OCT (left, Panel A, image field 10x10mm). Post-coagulation photograph (right, Panel B)

Movie S1 (separate file). Video of thermal relaxation in blood vessel with convective heat transfer incorporated into COMSOL

Movie S2 (separate file). Video of blood vessel coagulation in a large diameter blood vessel

Movie S3 (separate file). Video of blood vessel coagulation in artery vein pair before, during and immediately after laser coagulation. Corresponds to panels in Fig. 3.

**SI References**

1. Kimel S, Svaasand LO, Hammer-wilson M, et al. Differential Vascular Response to Laser Photothermolysis. *J Invest Dermatol*. 1994;103(5):693-700. doi:10.1111/1523-1747.ep12398548

2. Kimel S, Svaasand LO, Cao D, Hammer-Wilson MJ, Nelson JS. Vascular response to laser photothermolysis as a function of pulse duration, vessel type, and diameter: Implications for port wine stain laser therapy. *Lasers Surg Med*. 2002;30(2):160-169. doi:10.1002/lsm.10016

3. Nadort A, Kalk K, Leeuwen TG Van, Faber DJ. Quantitative blood flow velocity imaging using laser speckle flowmetry. 2016;(May 2015):1-10. doi:10.1038/srep25258

4. Maibier M, Reglin B, Nitzsche B, et al. Structure and hemodynamics of vascular networks in the chorioallantoic membrane of the chicken. *Am J Physiol Hear Circ Physiol 311*. 2016. doi:10.1152/ajpheart.00786.2015

5. Tawaza H. Measurement of respiratory parameters in blood of chicken embryo. *J Appl Physiol*. 1971;30(Jan 1971).

6. Katta N, Estrada AD, Mcelroy AB, et al. Laser brain cancer surgery in a xenograft model guided by optical coherence tomography. *Theranostics*. 2019;9(12):3555-3564. doi:10.7150/thno.31811

7. Jia Y, Tan O, Tokayer J, et al. Split-spectrum amplitude-decorrelation angiography with optical coherence tomography. *Opt Express*. 2012;20(4):4710-4725. doi:10.1364/OE.20.004710

8. Braaf B, Vermeer KA, Sicam VADP, van Zeeburg E, van Meurs JC, de Boer JF. Phase-stabilized optical frequency domain imaging at 1-µm for the measurement of blood flow in the human choroid. *Opt Express*. 2011;19(21):20886-20903. doi:10.1364/OE.19.020886

9. Davé DP, Milner TE. Doppler-angle measurement in highly scattering media. *Opt Lett*. 2000;25(20):1523-1525. doi:10.1364/OL.25.001523

10. Yang M-TT, Chih-Chung T-TCH-LL and F-YC and C-HY and C-KL and. Microvascular Imaging Using Swept-Source Optical Coherence Tomography with Single-Channel Acquisition. *Appl Phys Express*. 2011;4(9):97001. http://stacks.iop.org/1882-0786/4/i=9/a=097001.

11. Mariampillai A, Leung MKK, Jarvi M, et al. Optimized speckle variance OCT imaging of microvasculature. *Opt Lett*. 2010;35(8):1257-1259. doi:10.1364/OL.35.001257

12. Potsaid B, Baumann B, Huang D, et al. Ultrahigh speed 1050nm swept source / Fourier domain OCT retinal and anterior segment imaging at 100,000 to 400,000 axial scans per second. *Opt Express*. 2010;18(19):20029-20048. doi:10.1364/OE.18.020029

13. Hen CHIC, Ang RUKW. Optical coherence tomography based angiography [ Invited ]. *Biomed Opt Express*. 2017;8(2):2404-2422.

14. Chen Z, Milner TE, Srinivas S, et al. Noninvasive imaging of in vivo blood flow velocity using optical Doppler tomography. *Opt Lett*. 1997;22(14):1119-1121. doi:10.1364/OL.22.001119

15. Friebel M. Influence of oxygen saturation on the optical scattering properties of human red blood cells in the spectral range 250 to 2000 nm. *J Biomed Opt*. 2009;14(May):1-6. doi:10.1117/1.3127200

16. Friebel M. Determination of optical properties of human blood in the spectral range 250 to 1100 nm using Monte Carlo simulations with hematocrit-dependent effective. *J Biomed Opt*. 2006;11(June 2006):1-10. doi:10.1117/1.2203659

17. Jacques SL. Optical properties of biological tissues : a review Corrigendum : Optical properties of biological tissues : 2013. doi:10.1088/0031-9155/58/14/5007

18. Barton JK, Rollins A, Yazdanfar S, Pfefer TJ, Westphal V, Izatt JA. Photothermal coagulation of blood vessels : a comparison of high-speed optical coherence tomography and numerical modelling. 2001;46:1665-1678.

19. Tunnell JW, Wang L V, Anvari B. Optimum pulse duration and radiant exposure for vascular laser therapy of dark port-wine skin : a theoretical study. 2003;42(7):1367-1378.

20. Dai T, Pikkula BM, Tunnell JW, Chang DW, Anvari B. Thermal Response of Human Skin Epidermis to 595-nm Laser Irradiation at High Incident Dosages and Long Pulse Durations in Conjunction With Cryogen Spray Cooling : An Ex-Vivo Study. 2003;24(March):16-24. doi:10.1002/lsm.10183

21. Tunnell JW, Chang DW, Johnston C, et al. Effects of Cryogen Spray Cooling and High Radiant Exposures on Selective Vascular Injury During Laser Irradiation of Human Skin. 2003;139(June).

22. Tunnell JW. Selective Vascular Injuriy during cutaneous laser therapy. 2002.

23. Babilas P, Shafirstein G, Bäumler W, et al. Selective photothermolysis of blood vessels following flashlamp-pumped pulsed dye laser irradiation: In vivo results and mathematical modelling are in agreement. *J Invest Dermatol*. 2005;125(2):343-352. doi:10.1111/j.0022-202X.2005.23773.x

24. Katta N, Mcelroy A, Estrada A, Milner TE. Optical coherence tomography (OCT) guided smart laser knife for cancer surgery. In: *Proc.SPIE*. Vol 10054. ; 2017. https://doi.org/10.1117/12.2250699.
